# Mucus Is a Key Factor in Neisseria meningitidis Commensalism

**DOI:** 10.1128/mSphere.00777-19

**Published:** 2019-12-04

**Authors:** Melanie M. Callaghan, Joseph P. Dillard

**Affiliations:** a University of Wisconsin—Madison, Madison, Wisconsin, USA

**Keywords:** *Neisseria meningitidis*, commensal, mucus

## Abstract

The work presented by Audry et al. (M. Audry, C. Robbe-Masselot, J.-P. Barnier, B. Gachet, et al., mSphere 4:e00494-19, 2019, https://doi.org/10.1128/mSphere.00494-19) gives new insight into the interactions of Neisseria meningitidis and the human nasopharynx. Using an air interface tissue culture model of a polarized, mucus-secreting epithelium, Audry et al. demonstrate that N. meningitidis bacteria do not commonly invade epithelial cells. Rather, they are trapped in the mucus layer, where they are protected from dessication. In this model, meningicocci fail to elicit a pro-inflammatory immune response and show growth effects in response to another nasopharyngeal colonizer, Streptococcus mitis. These findings prompt new questions about pathobiont behaviors, the role of mucus in bacterium-host interactions, and modeling human infection.

## Abstract (precis)

The work presented by Audry et al. (M. Audry, C. Robbe-Masselot, J.-P. Barnier, B. Gachet, et al., mSphere 4:e00494-19, 2019, https://doi.org/10.1128/mSphere.00494-19) gives new insight into the interactions of Neisseria meningitidis and the human nasopharynx.

## COMMENTARY

Bacterial meningitis caused by Neisseria meningitidis is a worldwide health concern with a multitude of morbidities and high mortality rate. However, N. meningitidis is found frequently in the healthy human nasopharynx (1). Ample research into the bacterium’s interaction with the host has been pursued, and mechanisms of infection have been hot questions in the field for many years. As a result, many active virulence strategies including epithelial cell invasion and complex immune evasion have been studied in the laboratory (2). Yet the mystery remains unsolved—why does this “pathogen,” capable of causing debilitating disease, live as a commensal in 8 to 25% of people (1)?

Now, an innovative infection model employed by Audry et al. may have just opened the door to better understanding this conundrum (3). The pathogenic *Neisseria*—N. meningitidis and its close relative, Neisseria gonorrhoeae—are human restricted pathogens. This means that the task of studying how they infect their host is especially challenging, and development of relevant model systems is crucial. Cell culture is a popular approach, and these models have revealed aggressive virulence strategies of meningococci that illuminate the underpinnings of meningococcal disease (4). However, Audry et al. use “air interface culture” (AIC) to demonstrate commensal behavior of meningococci on a polarized bronchial epithelial cell line, Calu-3. In this model, the mucus-coated surface of the cells is exposed to air instead of covered with media, akin to the nasopharyngeal lumen. They demonstrate that under these conditions, N. meningitidis acts like a commensal; it does not interact with epithelial cells, does not use type IV pili, and does not induce an inflammatory response.

For starters, Audry et al. (3) show that without a liquid overlay, meningococci tend to reside in the mucus secretions of the Calu-3 cell layer. Adding medium over the mucus allows for growth uniformly across the cell culture model. In the epithelium’s infancy, the mucus secretions are spotty, and the bacterial cells are isolated to these islands of mucus in the AIC model. To put a finer point on this finding, N. meningitidis bacteria grown in the presence of dry mucus survive desiccation better than those grown without mucus. This is especially relevant to the nasopharyngeal space; in the host, bacterial cells are trapped in the mucus layer coating the epithelial surface, and the lumen to which they are exposed is full of air—a dry, dehydrating environment. Further investigation of the bacterium’s growth in this model revealed that meningococci remain in the mucus layer, failing almost completely to invade the epithelial cells even when they were treated with inflammatory cytokines that lower the cell’s resistance to invasion.

The data tell a story of a commensal meningococcus that resides harmlessly in a mucus overlay, and the authors probe this idea by comparing gene expression and inflammation in their model to the liquid overlaid system. Expression levels for 13 genes previously shown to be involved in colonization of mucosal surfaces were measured. Only four genes, *opaB* and *opaC* (adhesins), *nadA* (adhesin/invasin), and *fhbp* (factor H binding protein) were expressed differently in the two systems, with all four genes being expressed at lower levels in AIC (3, 5, 6). Additionally, pro-inflammatory cytokine measurements were lower in the AIC system. These findings solidly support the idea that meningococci reside in the mucus layer, protected and keeping a low profile.

The final question that Audry et al. (3) investigate is how various bacterial species that coexist in the human nasopharyngeal niche may influence one another. They test cocultures of meningococci with either Streptococcus mitis or Moraxella catarrhalis, neither of which thrive in this culture model on their own. Interestingly, S. mitis coinfection did enhance N. meningitidis growth in the AIC model. They demonstrate that S. mitis is capable of glycan remodeling in mucus and posit this (among others) as a potential mechanism of interaction between these bacteria that is relevant to survival in the host.

This study brings the AIC model to the forefront of research in meningococcal pathogenicity, opening the door for a slew of new questions to be addressed. Further exploration into the dichotomous nature of pathobionts is necessary for understanding these bacteria. If meningococci survive in the mucus without causing cellular damage or inflammation, what triggers the aggressive invasion seen in cases of meningococcal disease? Are specific components of mucus acting as signals, as has been observed in Pseudomonas aeruginosa (7)? Furthermore, time and time again studies show groundbreaking effects of host microbiota on health and disease states. The successful coculture and intriguing interactions that this study reports indicate a potential for microbiota studies, allowing for further elucidation of commensal and pathogenic microbial interactions in the nasopharyngeal space.

Finally, the successful use and novel findings resultant from the model raise questions regarding studies of other bacteria and mucosal surfaces. N. meningitidis is not the only commensal-turned-pathogen found in the nasopharynx; bacteria such as Streptococcus pneumoniae and Haemophilus influenzae both commonly colonize this niche as well (8). Utilization of the Calu-3 AIC model for study of these pathobionts may be similarly enlightening. Moreover, the exposed mucus bilayer of this model is relevant to many tissue culture models of lumenal infections. For example, N. gonorrhoeae is a close relative of N. meningitidis that infects mucosal surfaces such as the urethra, cervix, and pharynx. N. gonorrhoeae generally elicits a stronger inflammatory response than *N. meninigitidis*; however, gonorrhea infections are also often asymptomatic (2, 9). Could mucus help to explain this observation in a tissue culture model? Clearly, the implications that arise from this publication extend beyond both *N. meningitis* and the nasopharynx.

Audry et al. (3) make us question the “pathogenic” behavior of *Neisseria* species that do not always cause symptomatic infection, and a mucus layered tissue model may help answer these questions. On the whole, this study contributes significantly to the field of N. meningitidis-host interactions by demonstrating commensal-like behavior on a polarized bronchial epithelial tissue culture model, but more importantly, it paves the way for future research into the complex dynamics of “commensal” and “pathogenic” bacterial lifestyles in a simulated host environment.
